# Discovery and Characterization of an Atypical Crustin Antimicrobial Peptide from *Pollicipes pollicipes*

**DOI:** 10.3390/md22120526

**Published:** 2024-11-22

**Authors:** Wei Zhang, Liumi Wei, Pengyu Chen, Biao Ning, Junjian Wang, Peng He, Chenjing Shang, Dahui Yu

**Affiliations:** 1 Guangxi Key Laboratory of Marine Environmental Change and Disaster in Beibu Gulf, Beibu Gulf University, Qinzhou 535011, China; zhangwei9403@bbgu.edu.cn (W.Z.); wangjunjian@bbgu.edu.cn (J.W.); 2 Guangxi Key Laboratory of Beibu Gulf Marine Biodiversity Conservation, Beibu Gulf University, Qinzhou 535011, China; wlmbbgu@163.com (L.W.); pearlydh@163.com (D.Y.); 3 Environmental Science, Shenzhen Engineering Laboratory for Marine Algal Biotechnology, Guangdong Provincial Key Laboratory for Plant Epigenetics, College of Life Sciences and Oceanography, Shenzhen University, Shenzhen 518060, China; py.chen@szu.edu.cn; 4 College of Marine Science BGU, Beibu Gulf University, Qinzhou 535011, China; 15977483865@163.com

**Keywords:** antimicrobial peptides, crustin, antibacterial mechanism, *Pollicipes pollicipes*, antibiotic

## Abstract

Crustins are a family of antimicrobial peptides (AMPs) that play a pivotal role in the innate immune system of crustaceans. The discovery of novel AMPs from natural sources is crucial for expanding our current database of these peptides. Here, we identified and characterized a novel member of the crustin family, named *Pp*Crus-SWD1, derived from *Pollicipes pollicipes*. *Pp*Crus-SWD1 consists of 138 amino acids and contains eight cysteine residues that form a conserved ‘four-disulfide core’ structure. Our recombinant *Pp*Crus-SWD1 (r*Pp*Crus-SWD1) exhibited potent inhibitory activity against three Gram-positive bacteria (*Staphylococcus aureus*, *Bacillus* sp. T2, and *Streptococcus agalactiae*) and six Gram-negative bacteria (*Aeromonas hydrophila*, *Escherichia coli*, *Vibrio anguillarum*, *Vibrio alginolyticus*, *Vibrio parahemolyticus*, and *Acinetobacter* sp. L3), with minimum inhibitory concentrations ranging from 16 to 64 μM. Furthermore, r*Pp*Crus-SWD1 demonstrated binding affinity towards both bacteria and pathogen-associated molecular patterns (PAMPs), and damaged bacterial barrier. Additionally, it effectively inhibited alkaline protease activity in *S. aureus* and *V. alginolyticus* strains. These findings highlight the potential utility of this newly discovered crustin as an effective alternative to antibiotics.

## 1. Introduction

The development of antimicrobial peptides (AMPs) is imperative in response to the escalating global crisis of antibiotic resistance [1]. With the diminishing efficacy of conventional antibiotics against numerous pathogens, there arises an urgent demand for innovative antimicrobial agents [2]. AMPs, being naturally occurring peptides with broad-spectrum activity, present a promising solution. Not only do they possess the potential to combat multidrug-resistant bacteria, they also exhibit distinctive mechanisms of action that mitigate the likelihood of resistance development [3,4]. Moreover, through engineering modifications, AMPs can be enhanced in terms of stability, specificity, and efficacy, thus rendering them valuable candidates for therapeutic applications in both human and veterinary medicine [3,5].

Crustaceans, due to their extensive diversity and wide distribution in marine and freshwater environments, primarily rely on humoral and cellular innate immune responses for combating pathogens. AMPs derived from crustaceans exhibit structural and genetic diversity, making them valuable templates for the development of novel antimicrobial agents [6,7]. Since the initial discovery of a crustacean AMP in *Carcinus maenas* in 1996 [8], about 70 crustacean AMPs have been documented in the Antimicrobial Peptide Database3 (APD3, http://aps.unmc.edu/AP/, accessed on 1 August 2024). Given the abundant biodiversity of crustaceans, new antibacterial peptides continue to be identified annually [9,10].

**Figure 1:**
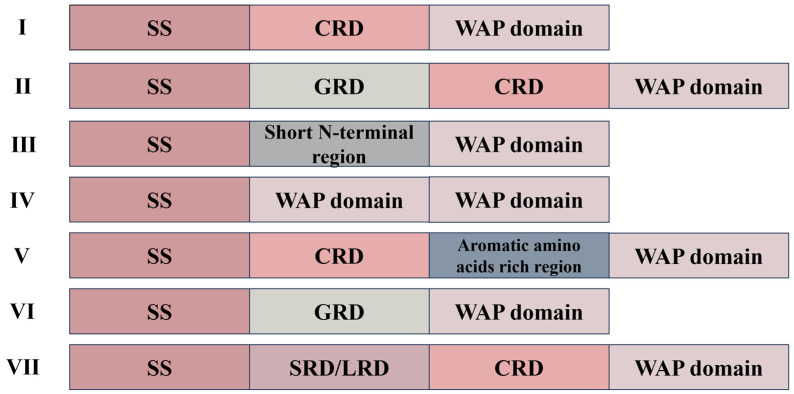
Schematic representation (not to scale) of the structural organization of the four crustin types (I to VII) found in crustaceans. CRD, cysteine-rich region; GRD, glycine-rich region; SRD, serine-rich region; LRD, leucine-rich region; WAP domain, whey acidic protein domain; SS, signal peptide.

Crustins, found in crustaceans, are cationic AMPs with molecular weights ranging from 6 to 22 kDa. They possess twelve conserved cysteine residues, of which eight form a characteristic whey acidic protein (WAP) domain. The WAP domain is characterized by a four-disulfide bond core structure located at the C-terminus and is involved in various functions such as antimicrobial activity and proteinase inhibition [11,12]. While the C-terminal WAP domain remains highly conserved, the N-terminal region exhibits significant variability in both length and amino acid sequence [13,14]. Based on differences in the N-terminal sequence, crustins were initially classified into seven types (Figure 1) [15]. Type I crustins include a cysteine-rich region preceding the WAP domain. Type II crustins feature [16,17] a glycine-rich region situated between the cysteine-rich region and the signal peptide. Type III crustins lack cysteine-rich region and glycine-rich region but instead have a proline-rich region along with the WAP domain [18]. Type IV crustins possess two WAP domains [19]. Type V crustin has an aromatic amino acid-enriched region between the WAP domain and the cysteine-rich domain [15]. Type VI crustin consists of a signal peptide followed by a glycine (Gly)-rich region and then the WAP domain [20]. Lastly, type VII crustin includes a signal peptide followed by a serine/leucine (Ser/Leu)-rich region and finally a WAP domain [21]. Although all seven types of crustins exhibit antimicrobial activity, their diverse structures result in variations in their antibacterial effects. Research suggests that crustins may inhibit bacterial growth through protease inhibitory or cell membrane damage [22,23]. However, research on crustins mostly focused on decapod crustaceans, there are very few studies on crustins from non-decapod crustaceans.

*Pollicipes pollicipes* is a member of barnacles [24]. Barnacles in the same sea area often exhibit higher levels of pathogenic microorganisms compared to shellfish, yet no reports have indicated mortality caused by these pathogens in barnacles [23,25]. Furthermore, barnacles possess the ability to cleanse biofilms in colonized environments [26]. Based on these observations, we hypothesized that barnacles may contain AMPs with potent antimicrobial activity. To identify novel crustins in *P. pollicipes*, we constructed a database of crustin protein sequences and compared them with those available in the NCBI database, leading us to discover an atypical member of the crustin family specific to *P. pollicipes*. Through recombinant expression in *Escherichia coli*, we successfully obtained a pure crustin protein without any purification tags, which was named recombinant *Pp*Crus-SWD1 (r*Pp*Crus-SWD1). In order to further explore the potential application of *Pp*Crus-SWD1 within the aquaculture industry, we evaluated its antibacterial activity and investigated its possible mechanism against pathogens.

## 2. Results

### 2.1. Sequence and Structure Characterization of PpCrus-SWD1

The amino acid sequence of *Pp*Crus-SWD1 (NCBI access number: XP_037090548.1) contains 138 amino acids with a calculated molecular weight of 14.33 kDa and a predicted pI of 8.10 by ProtParam. The CDS sequence encoding *Pp*Crus-SWD1 is 414 bp. The mature peptide of *Pp*Crus-SWD1 has 115 amino acids and a molecular weight of 12.19 kDa. The CDS sequence encoding the mature peptide of *Pp*Crus-SWD1 is 345 bp. Subcellular localization prediction using two tools indicates that *Pp*Crus-SWD1 is likely to be localized extracellularly.

**Figure 2:**
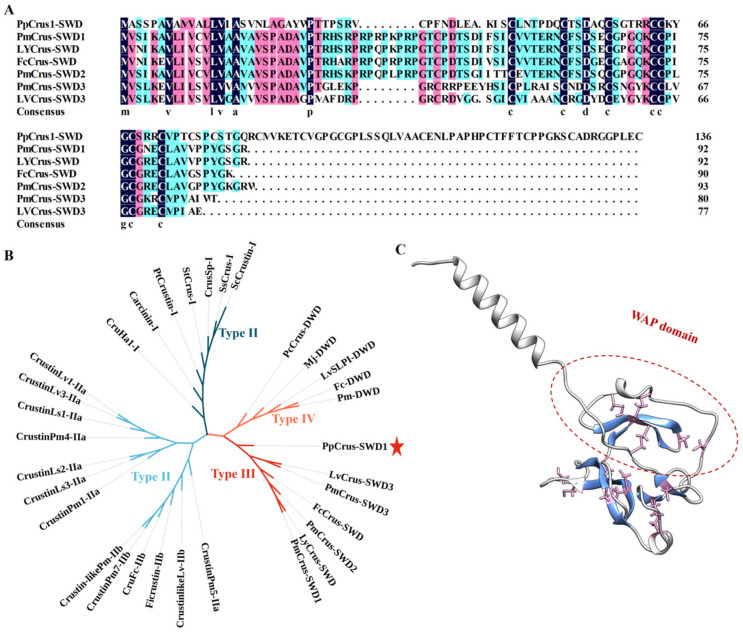
Sequence alignments and phylogenetic and structural analysis of *Pp*Crus-SWD1. (**A**) Alignment of *Pp*Crus-SWD1 with type III crustins (shown in Supplementary Table S2). The consensus residues are shaded black; the residues that are 100% identical among the aligned sequences are shaded blue; the residues that are ≥75% identical among the aligned sequences are shaded pink, the residues that are ≥50% identical among the aligned sequences are shaded cyan (**B**) Phylogenetic analysis of *Pp*Crus-SWD1 (red star) homologs. The phylogenetic tree was constructed with MEGA-X using the neighbor-joining method. Numbers beside the internal branches indicate bootstrap values based on 1000 replications. The crustins’ information used to construct the evolutionary tree is in Supplementary Tables S3 and S4. (**C**) The predicted structure of *Pp*Crus-SWD1 was built using Alphafold2. The cysteines in the WAP domain are shown in *Pink*, and the β-pleated sheet is shown in blue.

The N-terminus of *Pp*Crus-SWD1 contains a signal peptide (residues 1 to 23) and is followed by a WAP domain, which can form a ‘four-disulfide core’ structure through the formation of disulfide bonds between Cys33–Cys65, Cys44–Cys69, Cys52–Cys64, and Cys58–Cys73 (Figure 2A,C). These characteristics are consistent with type III crustin. Protein BLAST analysis revealed that *Pp*Crus-SWD1 shares similarity with *Urocitellus parryii* WAP four-disulfide core domain protein 5 (GenBank accession No. XP_026264141.1, percent identity is 37.84%, Supplementary Table S1). Compared to other type III crustins, *Pp*Crus-SWD1 has an extended sequence following the WAP domain in the C-terminal region (Figure 2A,C). This long C-terminal segment contains 10 cysteines that form four additional disulfide bonds (Cys77–Cys86, Cys80–Cys108, Cys117–Cys128, and Cys122–Cys137). Phylogenetic analysis revealed that *Pp*Crus-SWD1 was classified within the clade of type III crustins (Figure 2B). The predicted protein structure of *Pp*Crus-SWD1 exhibited a combination of random coils, two α-helices, and three β-pleated sheets (Figure 2C).

### 2.2. Recombinant Expression and Purification

**Figure 3:**
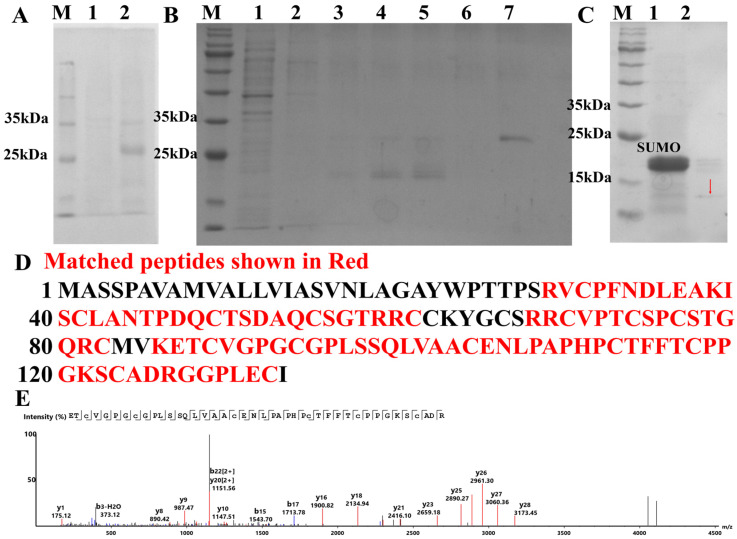
The acquisition process and MS spectrum analysis of r*Pp*Crus-SWD1. (**A**) SDS-PAGE analysis of recombinant *Pp*Crus-SWD1 (r*Pp*Crus-SWD1) expressed with a SUMO tag in *E. coli*. Lane M, protein marker; lane 1, total protein obtained from *E. coli* without induction; lane 2, total protein obtained from *E. coli* with IPTG induction. (**B**) His-SUMO-*Pp*Crus-SWD1 purified with nickel column chromatography. Lane M, protein marker; lane 1, protein not caught by the nickel column; lane 2, equilibration buffer; lane 3, eluent with 20 mM Imidazole; lane 4, eluent with 50 mM Imidazole; lane 5, eluent with 100 mM Imidazole, lane 6, eluent with 200 mM Imidazole; lane 7, eluent with 500 mM Imidazole. (**C**) SDS-PAGE analysis of r*Pp*Crus-SWD1 without SUMO tag. Lane M, protein marker; lane 1, His-SUMO-*Pp*Crus-SWD1 after treatment with SUMO enzyme; lane 2, r*Pp*Crus-SWD1 obtained after removing SUMO tag; the red arrow points to the band of r*Pp*Crus-SWD1. (**D**) Alignment of mass spectrometry results with r*Pp*Crus-SWD1 sequence. (**E**) MS spectrum of “ETCVGPGCGPLSSQLVAACENLPAPHPCTFFTCPPGKSCADR”. The red, blue, and black lines are the y ions, b ions, and noise signals detected by mass spectrometry, respectively.

Using pSmartI as the vector template, the mature peptide of *Pp*Crus-SWD1 was fused to the C-terminus of the His-SUMO tag. The SDS-PAGE analysis (Figure 3A) revealed distinct differences in band patterns between the 25 kDa and 35 kDa proteins expressed by the bacteria before and after IPTG induction. This band corresponds to the expected size of the His-SUMO-*Pp*Crus-SWD1 fusion protein, which consists of a His-SUMO tag (about 18 kDa) and a mature peptide of *Pp*Crus-SWD1 (12.19 kDa). The His-SUMO-*Pp*Crus-SWD1 can be eluted from the Ni-column using gradient elution imidazole eluent. 500 μM is the optimal elution concentration (Figure 3B, lane 7). After treatment with SUMO enzyme, recombinant *Pp*Crus-SWD1 (r*Pp*Crus-SWD1) without the His-SUMO tag was obtained and characterized on SDS-PAGE as under 15 kDa (Figure 3C).

### 2.3. Liquid Chromatography and Mass Spectrometry (LC-MS) Identification Results

Using LC-MS to analyze the amino acid sequence of r*Pp*Crus-SWD1, as shown in Figure 3E, four peptides were detected in total, and the amino acid coverage reached 68.84%. This demonstrates that through prokaryotic expression of the fusion protein, the tag was effectively removed by the SUMO protease, and r*Pp*Crus-SWD1 without foreign amino acids was obtained.

### 2.4. Antimicrobial Activity of rPpCrus-SWD1

The antibacterial activity of r*Pp*Crus-SWD1 was tested against two Gram-positive and four Gram-negative species by measuring the minimum inhibitory concentration (MIC) of each bacterium. As shown in Table 1, r*Pp*Crus-SWD1 exhibited apparent inhibitory activities against both Gram-positive and Gram-negative bacteria. For the Gram-negative bacteria, a concentration of 32 μM of r*Pp*Crus-SWD1 effectively inhibited the growth of *V. alginolyticus*, *V. parahemolyticus*, *E. coli*, and *A. hydrophila*. Compared to the Gram-negative bacteria, r*Pp*Crus-SWD1 had a lower MIC value for *S. aureus* and *Bacillus* sp. T2; both required a concentration of 16 μM.

### 2.5. Results of Molecular Dynamics (MD) Simulations 

**Figure 4:**
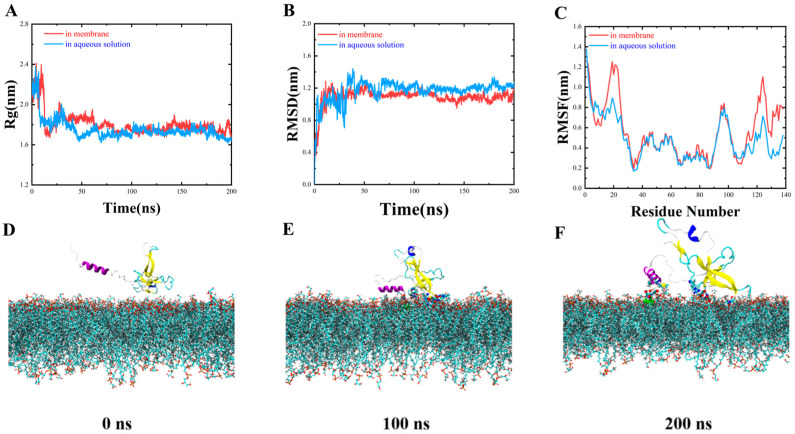
Molecular dynamics simulation. (**A**) The radii of gyration (Rg) of *Pp*Crus-SWD1 in aqueous solution and membrane. (**B**) The root–mean–square distances (RMSDs) of *Pp*Crus-SWD1 in aqueous solution and membrane. (**C**) The root–mean–square fluctuations (RMSFs) of *Pp*Crus-SWD1 in aqueous solution and membrane. (**D**) *Pp*Crus-SWD1 and membrane binding simulation (initial stage). (**E**) *Pp*Crus-SWD1 and membrane binding simulation (100 ns). (**F**) *Pp*Crus-SWD1 and membrane binding simulation (200 ns).

The root-mean-square distance (RMSD) and radius of rotation (Rg) of *Pp*Crus-SWD1 in aqueous solution and membrane are shown in Figure 4A,B. The RMSD results show that *Pp*Crus-SWD1 gradually reaches equilibrium after 120 ns in both water and membrane systems. The average Rg of *Pp*Crus-SWD1 is 1.72 nm in aqueous solution and 1.74 nm in the membrane, suggesting that *Pp*Crus-SWD1 is more extended when embedded within a membrane environment. In Figure 4C, the root–mean–square fluctuations (RMSFs) for both systems are presented; the β-sheet exhibits an RMSF value around 0.3 nm, indicating its rigidity while loop zones exhibit different degrees of flexibility. Specifically, the SER59-ARG62 region within the WAP domain initially contacts with POPG membranes followed by nearby amino acids (Figure 4D–F).

### 2.6. Microorganism-Binding Activity of rPpCrus-SWD1

**Figure 5:**
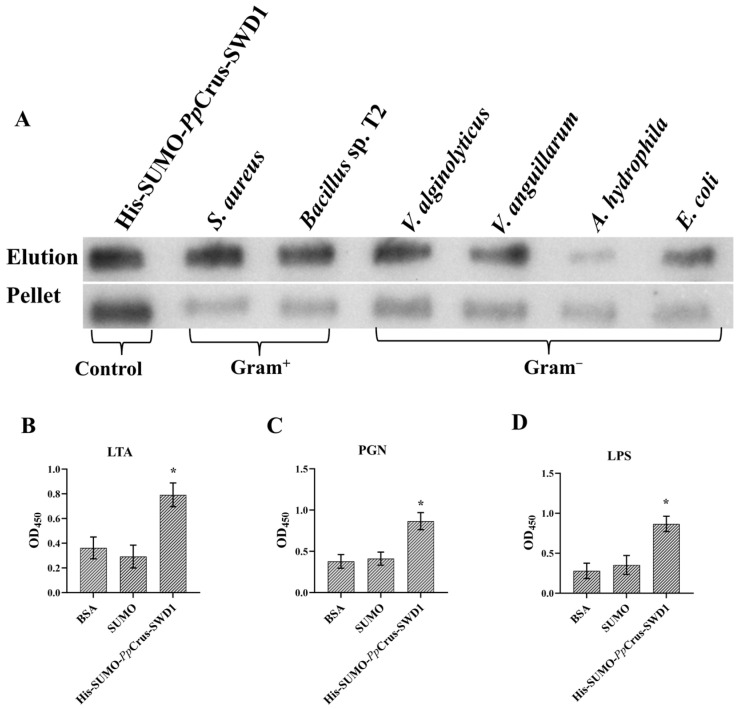
Microorganism-binding activity and PAMP-binding activity of r*Pp*Crus-SWD1. (**A**) Microorganism-binding activity. r*Pp*Crus-SWD1 was detected by Western Blot assay after treatment with bacteria (Gram^+^ and Gram^−^). r*Pp*Crus-SWD1 was taken as a positive control. Up panel, elution fractions; bottom panel, final pellet fractions. (**B**) Lipoteichoic acid binding activity. (**C**) Peptidoglycan binding assay. (**D**) Lipopolysaccharide binding assay. r*Pp*Crus-SWD1 was detected by ELISA. r*Pp*Crus-SWD1 was taken as a positive control. SUMO tag was taken as a negative control. LTA, Lipoteichoic acid; PGN, Peptidoglycan; LPS, Lipopolysaccharide. *, Compared with control (0 μM), *p*-value < 0.01.

Using His-SUMO-*Pp*Crus-SWD1 containing his tag for microorganism-binding assay. The negative control SUMO protein cannot bind to bacteria (Supplementary Figure S1). The result indicated that r*Pp*Crus-SWD1 binds to two Gram-positive bacteria (*S. aureus* and *Bacillus* sp. T2) and four Gram-negative bacteria (*A. hydrophila*, *E. coli*, *V. anguillarum*, and *V. alginolyticus*). These findings suggest that r*Pp*Crus-SWD1 may interact with bacterial cells (Figure 5A), contributing to its antibacterial activity.

### 2.7. Pathogen-Associated Molecular Patterns Binding (PAMP) Activity of rPpCrus-SWD1

Using His-SUMO-*Pp*Crus-SWD1 for PAMP-binding assay, the binding affinity of r*Pp*Crus-SWD1 to PAMP (Lipoteichoic acid, LTA; Peptidoglycan, PGN; and Lipopolysaccharide, LPS) was assessed using ELISA. The findings indicated that r*Pp*Crus-SWD1 at a concentration of 10 μM exhibited binding to Lipoteichoic acid, Peptidoglycan, and Lipopolysaccharide (all of PAMP were present at a concentration of 100 μg·mL^−1^, Figure 5B–D). In contrast, the negative control SUMO protein demonstrated no discernible binding to Lipoteichoic acid, Peptidoglycan, and Lipopolysaccharide.

### 2.8. Effects of rPpCrus-SWD1 on the Bacterial Morphology

**Figure 6:**
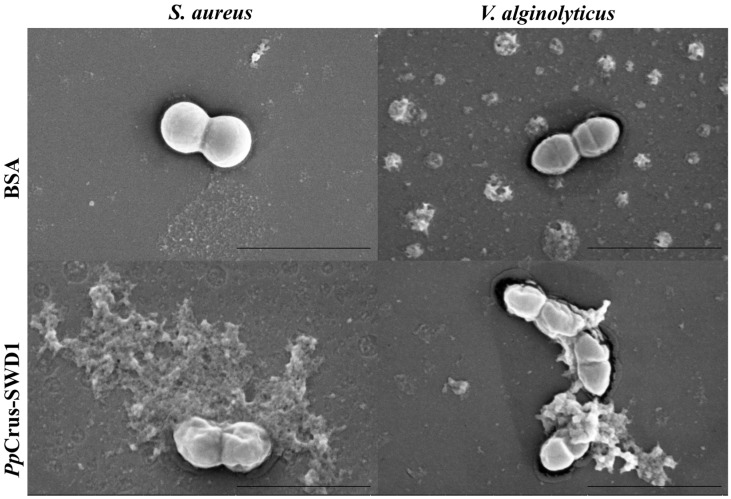
Morphological and cell membrane integrity changes of the bacterial cells treated with r*Pp*Crus-SWD1. Morphological changes of the bacterial cells treated with r*Pp*Crus-SWD1. About 10^6^ CFU·mL^−1^ bacteria were incubated with 2 × MIC of r*Pp*Crus-SWD1 for 2 h and observed under scan electron microscopy. PBS was used as a control. The scales are 2 μm. At the red arrow, the suspected bacterial content can be seen flowing out, and the cell surface shrinks.

The morphology of *S. aureus* and *V. alginolyticus*, following treatment with r*Pp*Crus-SWD1, exhibited irregular characteristics, characterized by a significant degree of cellular atrophy (Figure 6).

### 2.9. PI Staining

**Figure 7:**
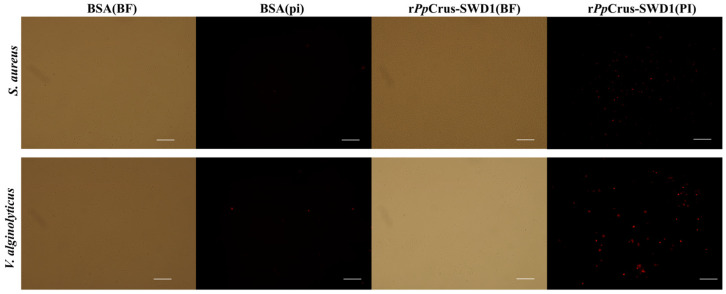
The effect of r*Pp*Crus-SWD1 on bacterial cell membrane integrity. About 1 × 10^6^ CFU·mL^−1^ bacteria were incubated with 2×MIC of rAaCrus1 for 2 h. PI: The cells were stained with PI and observed for PI uptake with a fluorescence microscope; BF: The bright field image. The scales are 50 μm.

Propidium iodide (PI) is impermeable to intact living cell membranes, but it can penetrate damaged cell membranes and stain the nucleus, as previously described [23]. PI staining revealed that treatment with r*Pp*Crus-SWD1 resulted in significant penetration of bacterial cells by PI in *S. aureus* and *V. alginolyticus* (Figure 7), indicating the ability of r*Pp*Crus-SWD1 to induce destabilization of bacterial cell membranes.

### 2.10. Protease Inhibition Activity and DNA Binding Activity

**Figure 8:**
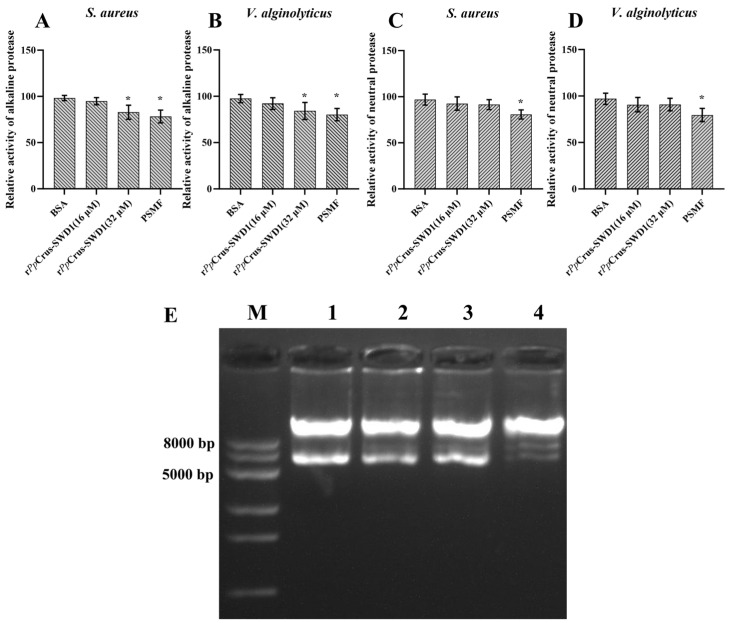
Ability to inhibit protease activity and DNA binding activity of r*Pp*Crus-SWD1. (**A**) Ability to inhibit the alkaline protease activity of *S. aureus*; (**B**) Ability to inhibit the alkaline protease activity of *V. alginolyticus*; (**C**) Ability to inhibit the neutral protease activity of *S. aureus*; (**D**) Ability to inhibit the neutral protease activity of *V. alginolyticus*; (**E**) Binding activity of r*Pp*Crus-SWD1 to plasmid DNA; lane M, marker; lane 1, negative control (BSA); lane 2, 8 μM r*Pp*Crus-SWD1 treatment; lane 3, 16 μM r*Pp*Crus-SWD1 treatment; lane 4, 32 μM r*Pp*Crus-SWD1 treatment; *, Compared with control (16 μM BSA), *p*-value < 0.01.

The proteinase-inhibiting activity of r*Pp*Crus-SWD1 was evaluated using *S. aureus* and *V. alginolyticus*. At a concentration of 32 μM, r*Pp*Crus-SWD1 exhibited significant inhibition against the alkaline protease activities of both *S. aureus* and *V. alginolyticus*, resulting in a reduction of 15.70% and 13.69% (Figure 8A,B), respectively. However, no notable inhibitory effect on the neutral protease activities of these bacteria was observed for r*Pp*Crus-SWD1 at any tested concentration up to 32 μM (Figure 8C,D, *p*-value > 0.05). Furthermore, even at the highest concentration tested (32 μM), r*Pp*Crus-SWD1 failed to affect DNA migration (Figure 8E).

### 2.11. Hemolytic Activity

**Figure 9:**
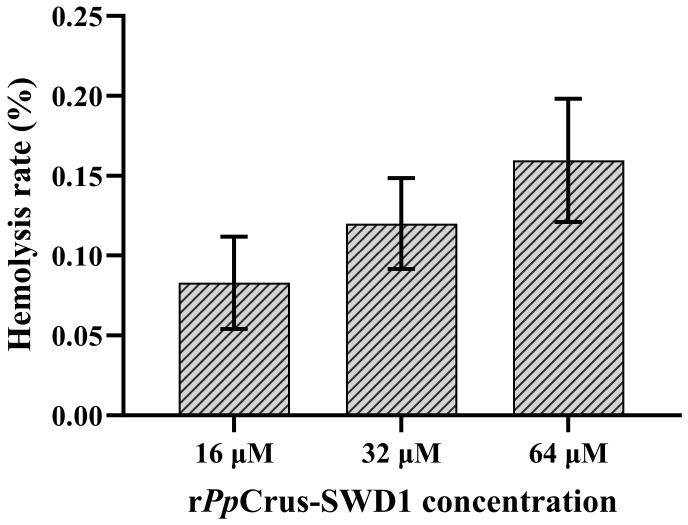
Hemolytic activity of rPpCrus-SWD1 on sheep red blood cells. The hemolysis rate of 1× PBS (pH 7.4) treatment was 0%, and that of 0.2% Triton X-100 treatment was 100%. There was no significant difference in hemolysis rate among the three concentration treatment groups (*p*-value > 0.05).

Hemolytic activity is an important indicator to evaluate the safety of antibacterial drugs. Our results demonstrated that as the concentration of r*Pp*Crus-SWD1 decreased (Figure 9), the hemolysis rate in sheep red blood cells also declined: from 15.91% at 64 μM, to 12.31% at 32 μM, and further to 8.78% at 16 μM. There was no significant difference in hemolysis rate among each concentration treatment groups (*p*-value > 0.05).

## 3. Discussion

The ocean is the cradle of life. Its unique ecological environment and biodiversity have nurtured numerous potent AMPs for the host to resist pathogen invasion, which provides new inspiration for novel antibacterial drug research and development [3,27].

Crustin is a class of AMPs widely found in marine crustaceans, which have broad-spectrum antibacterial activity and play a key role in the biodefense system [11,14]. Up to now, a wide range of crustins has been discovered in different types of crustaceans, such as amphipods, decapods, and isopods [14]. Typically, type I crustins primarily exhibit antibacterial properties against Gram-positive bacteria, while type II and III crustins are effective against both Gram-positive and Gram-negative bacteria [16,28,29]. The type II crustin in shrimp also plays a role in microbiome regulation [30]. In addition to their antibacterial effects, type III crustins also exhibit protease-inhibitory capabilities [29]. Type IV crustins possess either protease inhibitory activity or antibacterial properties, while the role of type V crustins remains unclear [31]. Type VI and VII crustins are currently only found in *Litopenaeus vannamei*; both have antibacterial and conditioning functions. Barnacles are widely distributed attached organisms in the world that show strong adaptability and immunity to complex marine environments [20,21]. This indicates that there may be AMPs in their bodies that have not been fully explored. Therefore, we chose crustin as the starting point for conducting a preliminary study on AMPs in *P. pollicipes.*

Following construction and comparative analysis of the protein database for crustins derived from *P. pollicipes*, we identified a new crustin composed of 138 amino acids. Its molecular weight is 14.33 kDa and it contains a WAP domain. Physicochemical property analysis and subcellular localization prediction indicated that it exhibits characteristics similar to crustins. BLAST and phylogenetic analysis revealed its close relationship with other type III crustins. Therefore, we designated it as *Pp*Crus-SWD1. The predicted structural model shows that *Pp*Crus-SWD1 (Figure 2), like other crustins, is composed of helices, strands, and coils [14,31]. In addition to the WAP domain shared by other type III crustins, *Pp*Crus-SWD1 also possesses an atypical long C-terminal region after the WAP domain. Based on the mature recombinant expression method developed in previous studies [23], we successfully obtained tag-free recombinant *Pp*Crus-SWD1 (r*Pp*Crus-SWD1). Results from LC-MS confirmed the robustness of our recombinant expression method and verified the correct production of r*Pp*Crus-SWD1. The minimum inhibitory concentration assay demonstrated that r*Pp*Crus-SWD1 exhibits broad-spectrum antibacterial activity against both Gram-positive and Gram-negative bacteria. These results indicate our successful identification of a novel crustin in the genome and proteome of *P. pollicipes*.

AMPs typically target multiple sites to eliminate bacteria or to impede bacterial growth [32,33]. Therefore, we conducted various experiments including molecular dynamics simulations, binding assays with bacteria and pathogen-associated molecular patterns (PAMP), scanning electron microscope observations, PI staining, and DNA binding tests to identify potential targets of *Pp*Crus-SWD1.

Binding to the target bacteria is a prerequisite for the AMP’s activity [34,35]. Through molecular dynamics simulations, we predict that *Pp*Crus-SWD1 can stably adhere to bacterial cell membranes and extract negatively charged lipid molecules using its cationic amino acids, resulting in damage to the cell membrane. The WAP domain is recognized as the crucial domain in crustin proteins [36,37], and in current research, the molecular dynamics simulations of *Pp*Crus-SWD1 also confirm the significance of the WAP domain. Molecular dynamics simulations revealed that the SER59-ARG62 region of the WAP domain in *Pp*Crus-SWD1 initially achieves stable binding with the cell membrane. The disulfide bonds in antimicrobial peptides maintain their stability [38]. MD simulation results indicate that the WAP domain in *Pp*Crus-SWD1 exhibits high stability, which may be correlated with the presence of multiple disulfide bonds within this region. In addition, MD simulation shows that several cationic amino acids within the WAP domain play a vital role in facilitating the transport of negatively charged POPG lipids. The results of the microorganism-binding assay demonstrate that *Pp*Crus-SWD1 can bind to a variety of bacteria, which supports the findings from molecular dynamics simulations predicting that *Pp*Crus-SWD1 can adsorb to the bacterial surface.

Certain AMPs are capable of utilizing lipoteichoic acid and peptidoglycan on the bacterial surface to exert antibacterial effects by traversing across the bacterial plasma membrane with the assistance of a polyanionic ladder formed by lipoteichoic acid [39,40]. Peptidoglycan on bacterial membranes attracts cationic AMPs to bind and insert into the membrane, ultimately leading to membrane leakage [22]. The binding assay for PAMP indicated that *Pp*Crus-SWD1 likely binds to bacteria through negatively charged lipoteichoic acid, peptidoglycan, and lipopolysaccharide, further supporting the results of MD simulations. Scanning electron microscopy (SEM) revealed atrophy in the bacterial cell surface after treatment with r*Pp*Crus-SWD1, suggesting possible discharge of cellular contents and indicating damage to the bacterial membrane. Furthermore, PI staining confirmed that r*Pp*Crus-SWD1 altered the permeability of bacterial cell plasma membranes. This may be attributed to r*Pp*Crus-SWD1 binding and interacting with lipoteichoic acid and peptidoglycan in the composition of the bacterial barrier. Consequently, we believe that targeting the bacterial cell barrier is crucial for the antibacterial activity exhibited by r*Pp*Crus-SWD1. Through its cationic properties, *Pp*Crus-SWD1 binds to the bacterial surface and interacts with its components, causing damage to the bacterial cell barrier and resulting in cell death.

Some AMPs target bacterial virulence factors such as lipopolysaccharides and proteases to mitigate their detrimental effects on the host [41,42]. During the process of bacterial infection, protease promotes the infection by hydrolyzing host proteins for various biological purposes [43,44]. Some members of the WAP domain protein family, including SLPI and elafin, have been shown to function as serine proteinase inhibitors and may also regulate pathogen-derived proteinases [45]. Additionally, certain type III crustins also exhibit inhibition of protease activity [46], with *Pp*Crus-SWD1 being closely related to type III crustin. Therefore, we undertook experiments to assess whether r*Pp*Crus-SWD1 acts as a protease inhibitor. At a concentration of 64 μM, r*Pp*Crus-SWD1 significantly inhibited alkaline protease activities in *S. aureus* and *V. alginolyticus* but had no significant effect on neutral protease activity. The function of protease activity inhibitors associated with the WAP domain is characterized by methionine (Met) adjacent to the second cysteine on the shear-able peptide [45]. In some crustaceans, Met is replaced by cationic and hydrophobic amino acids resulting in antimicrobial activity [11]. In the case of SWDPm2, Met is replaced by valine (Val) at the predicted cleavable peptide bond and exhibits both antimicrobial and antiprotease activity [29]. The substitution of Met with a leucine residue at a specific hydrophobic site in r*Pp*Crus-SWD1 likely contributes to the preservation of its protease inhibitory function, owing to the similar hydrophobic properties shared by these two amino acids.

Lipopolysaccharide functions as a virulence factor capable of inducing inflammation in host organisms [47]. Anti-lipopolysaccharide factors (ALFs) have the ability to inhibit the activation induced by bacterial endotoxins by binding to lipopolysaccharides [48]. Given that r*Pp*Crus-SWD1 also exhibits the ability to bind to lipopolysaccharides, we hypothesize that r*Pp*Crus-SWD1 may possess the function of inhibiting bacterial endotoxin activation. DNA is a target for some antimicrobial peptides, on the one hand penetrating bacteria and binding to DNA to affect the transcription process. On the other hand, it binds to DNA outside the bacterial cell and interferes with the horizontal transfer of bacterial virulence genes [49,50]. However, experimental findings indicate that r*Pp*Crus-SWD1 cannot bind to plasmid DNA. Therefore, bacterial DNA is not the target of its antibacterial action.

At MIC of *S. aureus* (16 μM), r*Pp*Crus-SWD1 demonstrated a hemolysis rate of less than 10%, indicating its limited cytotoxicity towards red blood cells at this concentration [51]. At the MIC for *V. anguillarum* (64 μM), the hemolysis rate of r*Pp*Crus-SWD1 was 15%, which is higher compared to *S. aureus* but still considered relatively low. These findings suggest that r*Pp*Crus-SWD1 holds promise as an antimicrobial peptide template with low destructive effects. Subsequently, we will conduct further toxicity assessment experiments and initiate in vivo animal studies on r*Pp*Crus-SWD1 in order to provide additional data support for the practical application of r*Pp*Crus-SWD1.

In summary, the proposed mechanism of *Pp*Crus-SWD1 involves electrostatic binding to bacteria, enabling the translocation of negative electric molecules across the bacterial membrane through its stable structure and cationic amino acids. Additionally, it interacts with lipoteichoic acid and peptidoglycan, causing membrane damage. Furthermore, by binding to lipopolysaccharides and inhibiting bacterial protease activity, *Pp*Crus-SWD1 may effectively alleviate the damage caused by bacteria to the host.

## 4. Materials and Methods

### 4.1. Bacterial Strains and Culture Conditions

The experimental bacteria included three Gram-positive strains and six Gram-negative strains, *S. agalactiae*, *S. aureus*, *Bacillus* T2, *E. coli*, *V. anguillarum*, *V. alginolyticus*, *A. hydrophila*, *V. parahemolyticus*, and *Acinetobacter* sp. L3. These strains were originally glycerobacteria and were stored at −80 °C. They were gifts from Professor Chaogang Wang [48] and Professor Xiaohui Cai [52]. Before the experiment began, bacteria were inoculated in a 2 mL medium and cultured at 37 °C at 200 rpm for 12 h to activate them. Among them, *S. agalactiae*, *S. aureus*, *Bacillus* sp. T2, *Escherichia coli*, *Acinetobacter* sp. L3, and *A. hydrophila* were activated by Luria-Bertan (LB) medium, and *V. parahemolyticus*, *V. anguillarum*, and *V. alginolyticus* were activated by Zobell Marine Broth 2216 (2216E) medium (HB0132, Haibo, Qingdao, China).

### 4.2. Prediction and Identification of Crustin Sequences

*P. pollicipes* genome information (NCBI access number: GCF_011947565.3) and 91 crustin sequences of *Amphibalanus amphitrite* (1), *Penaeus vannamei* (49), *Penaeus monodon* (23), and *Portunus trituberculatus* (18) were obtained from NCBI and our previous research. A local database was built using crustin sequences from other species by makeblastdb. BLASTP (e-value threshold: 1 × 10^−5^) was used to identify potential crustin sequences in Periwinkle pollicipes from a local database. The Protein BLAST algorithm available at NCBI (accessed on 1 August 2024; https://blast.ncbi.nlm.nih.gov/Blast.cgi) was utilized for sequence homology searches. SignalP 6.0 was used to predict signal peptides [53]. Multiple sequence alignments were generated using ClustalW (accessed on 1 May 2024; https://www.genome.jp/tools-bin/clustalw). These alignments were visualized with GeneDOC. A phylogenetic tree was constructed using the neighbor-joining method in MEGA-X, with 1000 bootstrap replicates to assess reliability (Mega Limited, Auckland, New Zealand). The tree was edited with ITOL 6.3.2 [54]. Alphafold2 was used to predict the 3D structure of *Pp*Crus-SWD1 [55]. Protein physicochemical properties were predicted using ProtParam (accessed on 25 May 2024; https://web.expasy.org/protparam/). Subcellular localization analysis of *Pp*Crus-SWD1 was performed using the online sites WoLF PSORT (https://www.genscript.com/wolf-psort.html, 1 August 2024) and Cell-PLoc (http://www.csbio.sjtu.edu.cn/bioinf/Cell-PLoc-2/, 1 August 2024).

### 4.3. Expression and Purification of Recombinant PpCrus-SWD1 (rPpCrus-SWD1)

Optimized based on previous research [23], the mature peptide of *Pp*Crus-SWD1 was fused to the C-terminus of a His-SUMO tag and expressed in *E. coli* BL21 (DE3) (TransGen Biotech, Beijing, China). The *Pp*Crus-SWD1 nucleotide sequence (NCBI accession number: XM_037234653.1) was optimized for the *E. coli* codon, and BamHI and XhoI restriction sites were added at both ends of the sequence. The 348 bp sequence of the mature peptide was chemically synthesized by General Biosystems (Chuzhou, China) and ligated into the pSmartI vector (containing His-sumo tag) via BamHI and XhoI sites. The resulting recombinant plasmid, named pSmartI-*Pp*Crus-SWD1 (Supplementary Figure S2A), had a total size of 5908 bp. Primers *Pp*Crus-SWD1 EF (5′-TTA AGA TTC TTG TAC GAC GG-3′) and *Pp*Crus-SWD1 ER (5′-TGC TAG TTA TTG CTC AGC GG-3′) were used for PCR amplification under the following conditions: initial denaturation at 95 °C for 3 min, followed by 35 cycles of 95 °C for 30 s, 51 °C for 30 s, and 72 °C for 1 min, with a final extension at 72 °C for 5 min. General Biosystems delivered the pSmartI-*Pp*Crus-SWD1 plasmid as a lyophilized powder. Transformation into *E. coli* BL21 (DE3) was achieved using the heat shock method, and positive clones were selected on solid LB agar containing kanamycin (50 µg/mL). Positive single clones confirmed by PCR and sequencing were used for recombinant protein production. The positive signal clones were inoculated in an LB medium containing kanamycin, and the bacterial solution was shaken at 200 rpm at 37 °C for overnight incubation. The next day, the bacterial liquid cultured overnight was added to fresh LB medium at a volume ratio of 1:100, the bacterial liquid was shaken at 37 °C at 200 rpm, and cultured to OD600 = 0.6. Recombinant protein expression was induced by the addition of isopropyl-β-d-thiogalactoside (IPTG) to an ultimate concentration of 0.5 mM at 16 °C for 12 h.

The cells were lysed by TieChui *E. coli* Lysis Buffer and centrifuged at 4 °C for 30 min at 10,000 rpm to separate the supernatant and pellet. Crude proteins from uninduced and induced cells were extracted for 12% SDS-PAGE analysis. The His-SUMO-*Pp*Crus-SWD1 protein present in the supernatant was purified using Ni-column affinity chromatography. The purified His-SUMO-*Pp*Crus-SWD1 protein was dialyzed against 1× PBS for 24 h at 4 °C. To completely remove the His-SUMO tag, 1 unit of SUMO protease (purchased from General Biosystems) was added and incubated at 4 °C for 6 h. The His-SUMO tag was retained by the Ni-column, while the unbound fraction was collected as the r*Pp*Crus-SWD1 protein solution without the His-SUMO tag. The purified r*Pp*Crus-SWD1protein was analyzed by SDS-PAGE, and its concentration was determined using the BSA Protein Assay Kit (Beyotime, Shanghai, China) following the manufacturer’s instructions. The protein solution was then aliquoted, lyophilized, and stored at −80 °C as a powder.

### 4.4. Identification of PpCrus-SWD1 by Liquid Chromatograph Mass Spectrometer (LC-MS)

This part is consistent with our previous research. The r*Pp*Crus-SWD1 protein band from SDS-PAGE was excised and placed in a 1.5 mL Eppendorf tube. It was decolorized overnight at 37 °C with 50% acetonitrile and shaking at 200 rpm. After washing with pure acetonitrile to whiten and harden the gel, the supernatant was discarded, and trypsin was added along with ammonium bicarbonate. The mixture was incubated at 37 °C for 16 h. Following trypsin digestion, the solution was transferred to a new 1.5 mL Eppendorf tube. An extraction solution (1 part water to 4 parts acetonitrile with 0.5% formic acid) was added, and the mixture was sonicated for 10 min, centrifuged, and vacuum-dried for 4 h to obtain the protein powder. The protein powder was dissolved in a sample solution (1 part water to 49 parts acetonitrile with 0.5% formic acid), vortexed, and centrifuged at 13,000× *g* for 15 min at room temperature. The solution was then analyzed by LC-MS (TRIPLETOF 5600+, AB SCIEX, Singapore) to identify r*Pp*Crus-SWD1.

### 4.5. Antimicrobial Activity of the rPpCrus-SWD1

To identify the minimum inhibitory concentration (MIC) of r*Pp*Crus-SWD1, we used a modified Clinical and Laboratory Standards Institute (CLSI) microtiter plate assay. The bacteria were cultured until the OD600 reached 0.4, then diluted to 10^4^ CFU mL^−1^ with Mueller-Hinton Broth (MHB) medium (HB6232, Haibo, Qingdao, China). r*Pp*Crus-SWD1 was dissolved in 1× PBS, and 20 μL of the peptide was mixed with 80 μL of the diluted bacterial suspension in a 96-well plate. A series of final concentrations (64 μM, 32 μM, 16 μM, 8 μM, 4 μM, 2 μM, and 1 μM) was tested for the MIC, with 1× PBS serving as the negative control. The plate was incubated at 37 °C for 18 h, and the OD600 was measured at 0 h and 18 h. The lowest concentration at which no bacterial growth was observed was considered the MIC. The assay was conducted with three biological replicates and three technical replicates.

### 4.6. Molecular Dynamics (MD) Simulations

Given the importance of peptide–membrane interaction under computational simulations, a series of MD simulations were performed to study the interactions between *Pp*Crus-SWD1 and the membrane. *Pp*Crus-SWD1 was described with the CHARMM36 force field [56]. The membrane was composed of 324 POPG molecules with 162 POPG molecules in each layer. The solvent was represented by TIP3P water [57]. Two systems were simulated: *Pp*Crus-SWD1 along and *Pp*Crus-SWD1 membrane. A solvent box of 9.34 × 9.34 × 9.34 nm^3^ and 10.53 × 10.53 × 15.50 nm^3^ was built for the former and latter systems, respectively. In both systems, Na^+^ and Cl^−^ ions were added to maintain the system’s electrical neutrality. All molecular dynamics simulations were performed using GROMACS version 2023.3 [58], and VMD 1.9.3 [59] was used for trajectory visualization.

The cutoff for the short-range part of the Lennard–Jones and Coulomb interaction was set to 1.2 nm. The particle mesh Ewald algorithm [60,61] was used to compute the long-range part of the Coulomb interaction (with a grid space of 0.16 nm and a spline of order 4). The temperature was controlled at 310 K (above the phase transition temperature of POPG) using the V-rescale algorithm with a time constant of 1.0 ps [62]. The pressure was maintained at 1 bar with the C-rescale algorithm [63] in a semi-isotropic method where the time constant and compressibility were set to 5 ps and 4.5 × 10^−5^ (kJ·mol^−1^·nm^−3^)^−1^, respectively. All bonds were fixed using the LINCS algorithm [64], thus a time step of 2 fs could be applied. For each system, the energy was first minimized, followed by a 500 ps prebalance in the NVT and NPT system, followed by a 200 ns simulation of *Pp*Crus-SWD1 in the aqueous solution system and POPG membrane system.

### 4.7. Microorganism-Binding Assay

Western Blot experiment was used to detect the binding activity of r*Pp*Crus-SWD1 to bacteria. Briefly, the microorganisms (1 × 10^8^ CFU) in a 1.5 mL centrifuge tube were incubated in 200 μL of His-SUMO-*Pp*Crus-SWD1 protein (10 μM) by gentle rotation for 1 h at room temperature. The cells were collected and washed three times with 1× Tris Buffered Saline (TBS) and then resuspended. After the centrifugation at 10,000 rpm for 5 min, the bacteria and supernatant were directly loaded on SDS-PAGE for analysis, respectively*. His-*SUMO-*Pp*Crus-SWD1 was the positive control, and His-SUMO tag was the negative control. Finally, proteins were transferred onto a polyvinylidene fluoride membrane (PVDF), which was blocked with 5% skim milk in 1× TBST. The blot was incubated with HPR-Anti-His Antibody (1:30,000, Boyi, Changzhou, China). Detection was completed with the BeyoECL Plus (Beyotime, Shanghai, China), according to the manufacturer’s instructions.

### 4.8. Binding Assay for Pathogen-Associated Molecular Patterns

A modified enzyme-linked immunosorbent assay (ELISA) was used to test the binding of the recombinant protein to bacterial membrane surface components [65]. LTA and PGN powders were dissolved in 1× ELISA coating buffer to a concentration of 100 μg·mL^−1^. LTA and PGN were added to a 96-well plate (100 μL/well) and incubated overnight at 4 °C. The wells were washed three times with 1× PBST (pH 7.4). Five percent skim milk, (1× PBST, pH 7.4) was added to each well (100 μL/well) and incubated at 37 °C for 2 h. The wells were gently washed three times with 1× PBST (pH 7.4) to remove unbound non-fat milk. Test proteins (dissolved in 1× PBS, pH 7.4) at a concentration of 10 μM were added to the wells, with 10 μM BSA serving as a negative control. The plate was incubated at 37 °C for 1 h and then washed once with 1× PBST (pH 7.4). HPR-Anti-His Antibody (diluted 5000-fold in 1× PBST, pH 7.4, Boyi, Changzhou, China) was added to the wells and incubated at 37 °C for 1 h. After incubation, the wells were washed five times with 1× PBST (pH 7.4). One hundred microliters of TMB substrate solution was added to each well (100 μL/well) for color development, and then ELISA stop solution was immediately added to stop the reaction (200 μL/well). The absorbance at 450 nm was measured using an ELISA reader. This experiment was performed with three biological replicates and three technical replicates.

### 4.9. Electron Microscopy

The electron microscopy procedure was adapted from our previous studies [23]. *S. aureus* and *V. alginolyticus* were cultured in LB medium and 2216E medium to mid-log phase, respectively. The bacteria were then collected and resuspended to a concentration of 10^6^ CFU/mL in 1× PBS. They were incubated at 2 × MIC r*Pp*Crus-SWD1 concentration for 2 h on round coverslips in 24-well plates. After treatment, the cells were fixed with 5% glutaraldehyde in PBS (pH 7.4) for 10 h at 4 °C, followed by three washes with 1× PBS. Bacteria treated with BSA served as the control. The cells were dehydrated through a graded series of ethanol (30%, 50%, 70%, 80%, 90%, and 100%) for 10 min at each step at 4 °C [22]. The cells were critically point-dried (Hitachi-HCP, Hitachi, Tokyo, Japan), sputter-coated with gold (MC1000, Hitachi, Tokyo, Japan), and examined with scanning electron microscopy (SEM) (APREO S, Thermo Fisher Scientific, Eindhoven, The Netherlands).

### 4.10. PI Staining

*S. aureus* and *V. alginolyticus* were cultured as described above at their 2 × MIC concentration for 2 h. Then, the samples were stained with a PI staining kit (Sangon, Shanghai, China) according to the manufacturer’s instructions. The cells were observed with a fluorescence microscope (BX51, Olympus, Tokyo, Japan).

### 4.11. Protease Inhibition Assay

Activated *S. aureus* and *V. alginolyticus* were cultured at 37 °C and 200 rpm for 12 h. Cells were collected by centrifugation at 5000× *g* for 3 min, washed three times gently with 1× PBS (pH 7.4), and resuspended in the extraction buffer provided with the protease activity assay kit. Cells were subjected to 20 min of ultrasonic treatment (240 W power, 2 s on, 4 s off) on ice. After sonication, the samples were centrifuged at 10,000× *g* and 4 °C for 10 min to obtain the supernatant containing bacterial proteases. The negative control was 16 μM bovine serum albumin (BSA), and the positive control was 1 mM PMSF. Test proteins were adjusted to a concentration of 32 μM and 64 μM in 1× PBS (pH 7.4). The test protein (20 μL) was mixed with 20 μL of the protease-containing supernatant, and the mixture was incubated at 37 °C for 30 min. The final concentrations of the tested proteins were 16 μM and 32 μM, respectively. The enzyme activity was measured according to the manufacturer’s instructions for the alkaline protease activity assay kit (Mlbio, Shanghai, China) and the neutral protease activity assay kit (Mlbio, Shanghai, China), respectively. The alkaline protease activity and neutral protease activity of bacteria treated with BSA were set as 100%. This experiment was performed with three biological replicates and three technical replicates.

### 4.12. DNA Binding Assay

Agarose gel electrophoresis was employed to assess the binding activity of r*Pp*Crus-SWD1 bacterial plasmid DNA [66]. The virulence gene PirA (NCBI accession number: MH410659.1) of *Vibrio parahemolyticus* was chemically synthesized, and BamHI and XhoI restriction enzyme recognition sites were added to both ends. Subsequently, it was incorporated into the pSmartI vector and engineered as the pSmart-PirA plasmid (Supplementary Figure S2B). Then, in 20 μL binding buffer (1 mM EDTA, 5% glycerol, 20 mM KCl, 1 mM DTT, 10 mM pH8.0 Tris-HCl, 50 μg·mL^−1^ BSA), 20 μL test protein (concentrations of 16μM, 32 μM, 64 μM, respectively) was mixed with 0.5 μL pSmart-I plasmid DNA (total 400 ng), incubated at 37 °C for 1 h, and 64 μM BSA was used as control. After incubation, 5 μL mixture was mixed with 1 μL 6× nucleic acid sample buffer. Electrophoresis was performed in 1% agarose gel and 1× TAE buffer, the voltage was set to 120 V, and the results were observed after electrophoresis.

### 4.13. Hemolytic Activity Assay

The hemolytic activity of the recombinant protein on sheep red blood cells (SRBCs) was evaluated using a previously established method [67]. This procedure involved defibrinated sheep blood (SBJ-ST-S001, Sbjbio-Z, Nanjing, China) by washing it five times with sterile 0.85% saline at 4 °C, ensuring the supernatant was clear. Subsequently, centrifugation was employed to isolate the SRBCs, which were then resuspended to achieve a 4% dilution. For the assay setup, 150 μL of the SRBCs dilution was mixed with 150 μL of the test protein solution, achieving final concentrations of 16 μM, 32 μM, and 64 μM, in 1.5 mL microcentrifuge tubes. Negative (1× PBS, pH 7.4) and positive (0.2% Triton X-100) controls were included for comparison. Following incubation at 37 °C for 1 h, the mixtures underwent centrifugation, and the optical density (OD) of the supernatants was measured at 570 nm using a microplate reader. The hemolysis percentage was calculated using the formula:

$$Hemolysis\;\left(\%\right)=\frac{\left(A-A_{0}\right)}{\left(A_{100}-A_{0}\right)}\times 100\%$$

where A, A_0_, and A_100_ represent the OD values of the sample, negative control, and positive control, respectively. To ensure the reliability of the results, the experiment was conducted with three biological replicates, each with three technical replicates.

### 4.14. Statistical Analysis

Statistical analysis was conducted using GraphPad Prism 7.0 (GraphPad, San Diego, CA, USA). Significance was assessed with a one-way analysis of variance (ANOVA), and all data were reported as mean ± SD. *p*-value < 0.05 was deemed statistically significant.

## Figures and Tables

**Table 1:** The minimum inhibitory concentration (MIC) of *Pp*Crus-SWD1 and ampicillin against Gram-positive and Gram-negative bacteria.

Microorganism		Minimum Inhibitory Concentrations (μM)	
		r*Pp*Crus-SWD1	Ampicillin
Gram^+^	*S. aureus*	16	1
	*Bacillus* sp. T2	16	-
	*S. agalactiae*	32	4
Gram^−^	*V. alginolyticus*	32	-
	*V. anguillarum*	64	-
	*E. coli*	32	100
	*A. hydrophila*	32	50
	*Acinetobacter* sp. *L32*	64	-
	*V. parahemolyticus*	32	-

## Data Availability

All data generated or analyzed during this study are included in this published article, further inquiries can be directed to the corresponding author.

## Supplementary Materials

The following supporting information can be downloaded at: https://www.mdpi.com/article/10.3390/md22120526/s1, Figure S1: Microorganism-binding Assay of SUMO; Figure S2: Plasmid map; Table S1: Blast result of *Pp*Crus-SWD1 in NCBI; Table S2: Information on the crustins used in the sequence alignment of Figure 2A; Table S3: Information on type I and type II crustins used to construct the evolutionary tree; Table S4: Information on type III and type IV crustins used to construct the evolutionary tree; Supplement data: The nucleotide sequence of codon-optimized *Pp*Crus-SWD1.
