# Supplementary material for: Discovery and Characterization of an Atypical Crustin Antimicrobial Peptide from Pollicipes pollicipes
Source: Mar Drugs. 2024 Nov 22;22(12):526. doi: 10.3390/md22120526 (PMC11678330; doi:10.3390/md22120526)
Supplement: Supplementary file 1 [file marinedrugs-22-00526-s001.zip › marinedrugs-3330148-supplementary.pdf]

Table S1. Blast result of *Pp*Crus-SWD1 in NCBI.

Description	Total Score	Query Cover	E value	Per. ident	Acc. Len	Accession
WAP four-disulfide core domain protein 5-like [*Amphibalanus amphitrite*]	48.9	42%	0.001	42.37	169	XP_043233566.1
WAP four-disulfide core domain protein 5-like [*Amphibalanus amphitrite*]	49.3	42%	0.001	41.67	169	XP_043245360.1
WAP four-disulfide core domain protein 5 [*Marmota marmota marmota*]	44.7	52%	0.026	37.84	122	XP_048666682.1
WAP four-disulfide core domain protein 5 [*Urocitellus parryii*]	46.2	52%	0.011	37.84	147	XP_026264141.1
WAP four-disulfide core domain protein 5-like [*Marmota flaviventris*]	45.4	52%	0.013	37.84	127	XP_027809974.1
WAP four-disulfide core domain protein 5 [*Marmota monax*]	45.4	52%	0.014	37.84	122	XP_046289053.2
keratin-associated protein 10-6-like [A*mphibalanus amphitrite*]	56.2	76%	9.00E-06	37.04	556	XP_043233379.1
WAP four-disulfide core domain protein 5 [*Ictidomys tridecemlineatus*]	44.3	52%	0.043	36.49	122	XP_040131468.1
WAP four-disulfide core domain protein 12 [*Symphalangus syndactylus*]	46.6	52%	0.004	35.62	111	XP_055121779.1
antileukoproteinase-like isoform X1 [*Zalophus californianus*]	45.8	54%	0.012	33.33	142	XP_027476459.1
antileukoproteinase-like isoform X2 [*Zalophus californianus*]	45.8	54%	0.013	33.33	141	XP_027476461.1
antileukoproteinase-like isoform X3 [*Zalophus californianus*]	45.4	54%	0.017	33.33	140	XP_027476464.1
ELAF protein [*Corythaeola cristata*]	43.5	60%	0.028	33.33	74	NXC22193.1
elafin-like [*Rhea pennata*]	43.1	53%	0.037	33.33	76	XP_062445163.1
ELAF protein [*Indicator maculatus*]	43.1	60%	0.042	32.14	74	NXN10202.1
caltrin-like protein 2 [*Corapipo altera*]	44.7	55%	0.01	31.58	75	XP_027502173.1

Table S2. Information on the crustins used in the sequence alignment of Figure 2A.

Abbreviation	Explanation	NCBI Accession Number
*Pm*Crus-SWD1	*Penaeus monodon*	ACF28464.1
*LY*Crus-SWD	*Penaeus vannamei*	AAS38513.1
*Fc*Crus-SWD	*Penaeus chinensis*	ABN09668.1
*Pm*Crus-SWD2	*Penaeus monodon*	XP_037802161.1
*Pm*Crus-SWD3	*Penaeus monodon*	ACF28466.1
*LV*Crus-SWD3	*Penaeus vannamei*	ROT74235.1

Table S3. Information on the type I and type II crustinused to construct the evolutionary tree.

Abbreviation	Explanation	NCBI Accession Number
*Pp*crustin-I	*Portunus pelagicus*	AFN37210.1
*Sc*crustin-I	*Scylla serrata*	ADW11096.1
Crus*Sp*-I	*Scylla paramamosain*	ABY20727.1
Cru*Ha*1-I	*Hyas araneus*	ACJ06763.1
*Pt*Crustin-I	*Portunus trituberculatus*	ACM89167.2
Carcinin-I	*Carcinus maenas*	CAD20734.1
*Ss*Crus-I	*Scylla serrata*	ADW11096.1
Crustin*Lv*1-IIa	*Penaeus vannamei*	XP_027208055.1
Crustin*Lv*3-IIa	*Penaeus vannamei*	AAL36892.1
Crustin*Ls*1-IIa	*Penaeus setiferus*	AAL36897.1
Crustin*Ls2*-IIa	*Penaeus setiferus*	AAL36896.1
Crustin*Ls3*-IIa	*Penaeus setiferus*	AAL36898.1
Crustin*Pm*1-IIa	*Penaeus monodon*	ACQ66004.1
Crustin*Pm*4-IIa	*Penaeus monodon*	ACQ66005.1
CrustinPm5-IIa	*Penaeus monodon*	XP_037792256.1
Crustinlike*Lv*-IIb	*Penaeus vannamei*	XP_027224107.1
Cru*Fc*-IIb	*Penaeus chinensis*	XP_047502940.1
*Fi*crustin-IIb	*Penaeus indicus*	ACV84092.1
Crustin-like*Pm*-IIb	*Penaeus monodon*	ACT82963.1
Crustin*Pm*7-IIb	*Penaeus monodon*	ACT82963.1

Table S4. Information on the type III and type IV crustin used to construct the evolutionary tree.

Abbreviation	Explanation	NCBI Accession Number
*Pm*Crus-SWD1	*Penaeus monodon*	ACF28464.1
*LY*Crus-SWD	*Penaeus vannamei*	AAS38513.1
*Fc*Crus-SWD	*Penaeus chinensis*	ABN09668.1
*Pm*Crus-SWD2	*Penaeus monodon*	XP_037802161.1
*Pm*Crus-SWD3	*Penaeus monodon*	ACF28466.1
*LV*Crus-SWD3	*Penaeus vannamei*	ROT74235.1
*Lv*SLPI-DWD	*Penaeus vannamei*	QOL09973.1
Pm-DWD	*Penaeus monodon*	XP_037784226.1
Fc-DWD	*Penaeus chinensis*	ACY64754.1
Mj-DWD	*Penaeus japonicus*	ABW88999.1
PcCrus-DWD	*Procambarus clarkii*	XP_069170853.1

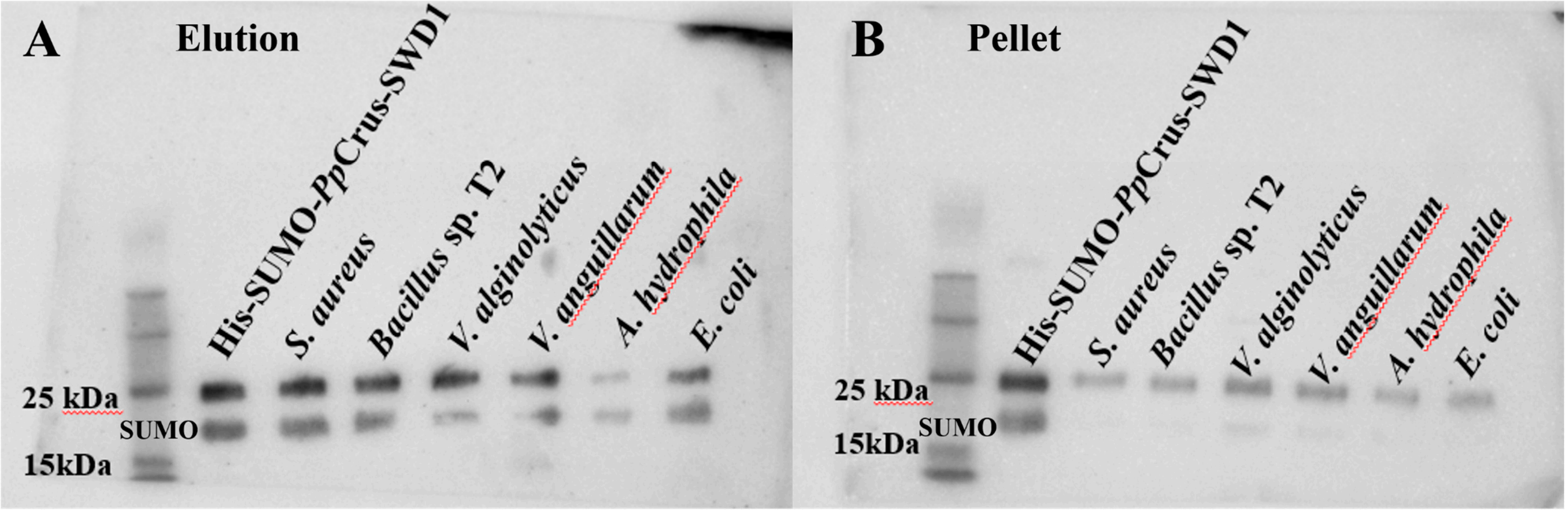

Figure S1. Microorganism-binding Assay of SUMO. (A) Elution fractions; (B) Final pellet fractions

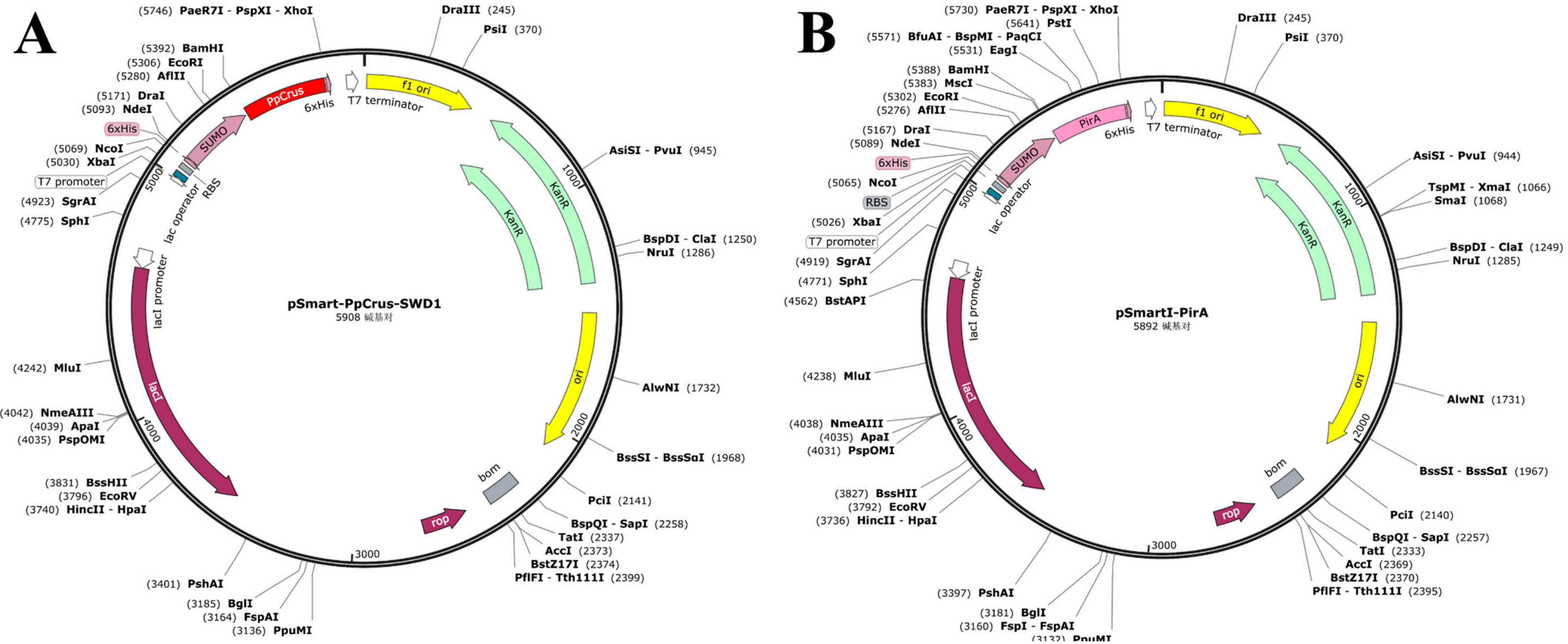

Figure S2. Plasmid map. (A)pSmartI-*Pp*Crus-SWD1 map; (B) pSmartI-PirA map .

Supplement data. The nucleotide sequence of codon-optimized *Pp*Crus-SWD1

CTACTGGCCGACCACCCCGAGTCGTGTTTGTCCGTTTAATGATCTGGAAGCCAAAATTAGCTGCCTGGCCAATACCCCGGATCAGTGTACCAGCGAT
GCACAGTGTAGTGGCACCCGTCGCTGCTGTAAATATGGCTGCAGCCGCCGTTGTGTTCCGACCTGCAGCCCGTGTAGCACCGGCCAGCGCTGTATGG
TGAAAGAAACCTGCGTTGGCCCGGGTTGCGGTCCGCTGAGCAGCCAACTGGTTGCAGCCTGCGAAAATCTGCCGGCACCGCATCCGTGTACCTTTTT
CACCTGTCCGCCGGGCAAAAGTTGCGCAGATCGTGGCGGTCCGCTGGAATGTATTTAA.
